# Pyocyanin Promotes Extracellular DNA Release in *Pseudomonas aeruginosa*

**DOI:** 10.1371/journal.pone.0046718

**Published:** 2012-10-08

**Authors:** Theerthankar Das, Mike Manefield

**Affiliations:** Centre for Marine BioInnovation (CMB), School of Biotechnology and Biomolecular Sciences (BABS), University of New South Wales (UNSW), Sydney, Australia; Universitätsklinikum Hamburg-Eppendorf, Germany

## Abstract

Bacterial adhesion and biofilm formation are both dependent on the production of extracellular polymeric substances (EPS) mainly composed of polysaccharides, proteins, lipids, and extracellular DNA (eDNA). eDNA promotes biofilm establishment in a wide range of bacterial species. In *Pseudomonas aeruginosa* eDNA is major component of biofilms and is essential for biofilm formation and stability. In this study we report that production of pyocyanin in *P. aeruginosa* PAO1 and PA14 batch cultures is responsible for promotion of eDNA release. A *phzSH* mutant of *P. aeruginosa* PAO1 that overproduces pyocyanin displayed enhanced hydrogen peroxide (H_2_O_2_) generation, cell lysis, and eDNA release in comparison to its wildtype strain. A Δ*phzA-G* mutant of *P. aeruginosa* PA14 deficient in pyocyanin production generated negligible amounts of H_2_O_2_ and released less eDNA in comparison to its wildtype counterpart. Exogenous addition of pyocyanin or incubation with H_2_O_2_ was also shown to promote eDNA release in low pyocyanin producing (PAO1) and pyocynain deficient (PA14) strains. Based on these data and recent findings in the biofilm literature, we propose that the impact of pyocyanin on biofilm formation in *P. aeruginosa* occurs via eDNA release through H_2_O_2_ mediated cell lysis.

## Introduction

Bacterial adhesion and biofilm formation represent significant and ongoing problems in contexts ranging from lethal bacterial infections to corrosion of engineered systems [1]–[3]. Biofilm persistence is attributed to a matrix of extracellular polymeric substances (EPS) made up of polysaccharides, proteins, lipids and extracellular DNA (eDNA) in which bacterial cells are embedded [4], [5]. A decade ago, Whitchurch and coworkers discovered that eDNA is a major component of biofilms formed by the opportunistic human pathogen *Pseudomonas aeruginosa* and is essential for biofilm formation and stability [6]. Since these seminal findings were made, it has been shown that eDNA promotes initial bacterial adhesion, cellular aggregation, biofilm strength and protection of bacterial cells in biofilms against antibiotics and detergents in a wide range of bacteria [7]–[13].

Extracellular DNA release occurs via lysis of subpopulations of bacterial cells mediated by diverse mechanisms. In *Staphylococcus epidermidis* eDNA release is mediated by the autolysin AtlE [14]. In *Enterococcus faecalis* eDNA release is mediated by gelatinase (GelE) and serine protease (SprE) [15]. In *Streptococcus pneumoniae* eDNA release is controlled by bacteriophage mediated cell lysis [16]. In other *Streptococcus* species pyruvate oxidase has been implicated in cell lysis and eDNA release via H_2_O_2_ generation [17], [18].

In *P. aeruginosa,* eDNA release is mediated through quorum sensing (QS) dependent mechanisms, involving *N*-acyl-L-homoserine lactones (AHL) and the *Pseudomonas* quinolone signaling (PQS) molecule, and through QS independent mechanisms via flagella and type IV pili [19], [20]. PQS in *P. aeruginosa* PAO1 triggers eDNA release in the early phase of planktonic culture through induction of prophage (19). In accordance, the mutant strain *pqsA* that lacks PQS and the *pqsL* mutant that overproduces PQS show low and high amounts of eDNA release respectively [19]. QS independent mechanisms, including phage induction [20], are responsible for eDNA release only in older PAO1 biofilms (>10 days) and not in growing planktonic cultures.

**Figure 1:**
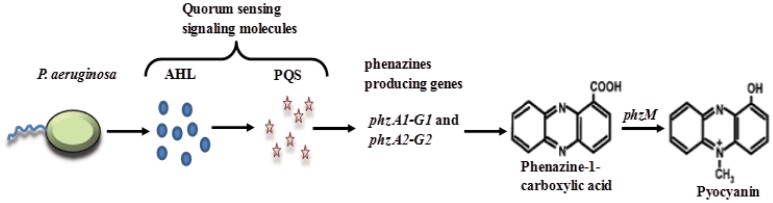
Schematic represents quorum sensing regulated expression of genes encoding phenazine and pyocyanin production. *P. aeruginosa* synthesizes Acylated Homoserine Lactones (AHLs) as their primary quorum sensing signaling molecules; AHLs further control the production of the secondary Pseudomonas Quinone Signaling (PQS) molecule. PQS regulates the synthesis of phenazine-1-carboxylic acid (PCA) through a set of primary phenazine producing genes *phzA1-G1* and *phzA2-G2.* PCA then converts into the derivative pyocyanin via *phzM*.

The complex QS circuitry in *P. aeruginosa* also regulates the production of phenazines, which are small, diffusible, electrochemically active compounds with a multitude of biological activities [21], [22]. Phenazine production in *P. aeruginosa* involves a step-by-step process starting with synthesis of the primary QS molecules (AHL) during exponential phase; followed by the secondary QS molecule (PQS) during late exponential phase and then PQS controlled expression of *phzA-G* operons resulting in production of phenazine-1-carboxylic acid (PCA). PCA is then modified to produce primarily pyocyanin (modification encoded by *phzM*) as shown in figure 1, but also phenazine-1-carboxamide (PCN, encoded by *phzH*) and 1-hydroxy phenazine (1-OHPHZ, encoded by *phzS*) [21], [23].

It has been demonstrated that altering the ratio of specific phenazine molecules produced, via activating or repressing/omitting specific *phz* genes, impacts on adhesion, biofilm formation and biocontrol activity [24], [25], [26]. Pyocyanin is also a virulence factor playing a major role in chronic lung infection in cystic fibrosis patients [22]. Pyocyanin production by *P. aeruginosa* followed by its interaction with molecular oxygen to form reactive oxygen species like hydrogen peroxide (H_2_O_2_) leads to changes in the redox balance of host cells causing cell injury and death [22]. Interestingly, it has been demonstrated that phenazines control gene expression and community behavior in divergent bacteria, including initial adhesion and the subsequent thickness of *Pseudomonas chlororaphis* biofilms [24], [25]. Additionally, it has been shown that phenazines control colony size and biofilm thickness in *P. aeruginosa* PA14 [27]. Based on these observations and the facts that pyocyanin generates H_2_O_2_ in Pseudomonads and H_2_O_2_ is involved in eDNA production in Streptococci, in this study we tested the hypothesis that pyocyanin is involved in eDNA release in *P. aeruginosa* via the generation of H_2_O_2_ in planktonic batch culture conditions.

## Materials and Methods

### Bacterial Species and Culture Conditions

All *P. aeruginosa* strains used in this study are listed in Table 1. Strains were plated onto LB agar plates and incubated overnight under aerobic conditions at 37°C. Single colonies from the agar plates were used to inoculate 20 ml cultures in LB medium (ph 7) for various growth times (0, 8, 16, 24, 72 and 120 h) at 30°C, 150 rpm. After growth, the *P. aeruginosa* strains were harvested and pelleted out by centrifugation at 6500 rpm (4912× *g*) for 5 min at 10°C. Where indicated the bacterial strains were also grown by adding 2 ml of bacterial cell free supernatant to 18 ml of LB medium (total 20 ml) for 24 h at 30°C, 150 rpm. In addition, experiments were done by growing bacterial strains in presence of exogenous addition of pyocyanin (Sigma-Aldrich) for 24 h at 30°C, 150 rpm.

**Table 1:** *P. aeruginosa* strains used in this study and their relevant phenazine producing characteristics.

*P.aeruginosa* strains	Phenazine production		Source
	PCA	Pyocyanin	
PAO1 Wildtype	+	+	this study
PAO1 *phzSH* mutant	+	++	this study
PA14 Wildtype	+	+	this study
PA14 ΔphzA-G	-	-	[24]

+ produce *basal level of pyocyanin*. ++ produce *elevated amount of pyocyanin*.

### Construction of *phzSH* Mutant Strain of *P. aeruginosa* PAO1

To alter the ratio of pyocyanin production in *P. aeruginosa* PAO1 we constructed the *phzSH* mutant that over produces pyocyanin. *P. aeruginosa* PAO1 *phzSH* mutant was constructed by a gene replacement technique, using plasmid pBR325 containing the disrupted *phzS* gene [28]. The disrupted *phzS* gene was mobilized into a *P. aeruginosa* PAO1 Δ*phzH* mutant [28] from *E. coli* GJ23 by conjugation. For conjugation approximately 10^9^ cells of *E. coli* GJ23 (donor) were mixed with 2×10^9^ cells of *P. aeruginosa* Δ*phzH* mutant (recipient) and incubated on a 0.45 µm nitrocellulose filter (Millipore) placed onto an LB plate at 37°C for 6–8 h. The filter was subsequently vortexed in 2 ml of phosphate buffered saline (PBS: 150 mM NaCl, 10 mM potassium phosphate) plated in 100 µl aliquots onto selective plates. Recombinations were selected on *Pseudomonas* Indicator Agar (PIA) plates containing 100 µg/ml gentamycin and 50 µg/ml tetracycline or 100 µg/ml of carbenicillin to counter select against the donor and *P. aeruginosa* PAO1 cells harbouring unintegrated pBR325. The double mutant was confirmed by PCR and sequencing using standard protocols.

### Quantification of eDNA in Bacterial Cell Free Supernatant

After centrifugation, supernatants were separated from bacterial pellets by simply transferring it into new tubes. In order to remove remaining bacteria, supernatants were again filtered using 0.22 µm Millipore filter units (Millipore). To further, ensure the filtered supernatants were free of bacterial cells, 50 µl of filtered supernatant was used to inoculate LB agar plates and incubated for 48 h under aerobic conditions at 37°C. No bacterial colony formation on LB agar plates was observed. The concentration of eDNA present in the filtered supernatant of various *P. aeruginosa* strains at various growth times was quantified by mixing 20 µl of supernatant with double stranded DNA quantifying fluorescent dye assay (dsDNABR) from Qubit, Invitrogen. The intensity of the fluorescent dye after binding with eDNA in the supernatant were quantified by using Qubit 2.0 Fluorometer (Invitrogen, Life Technologies, CA, USA). The measured amount of eDNA in the supernatants were plotted as µg/ml.

### Measurement of Pyocyanin and H_2_O_2_ in the Supernatant of *P. aeruginosa* Strains

Pyocyanin production by *P. aeruginosa* strains in the LB medium at various growth times was measured by taking 200 µl of bacterial cell free supernatant in 96-well microtiter plates and absorbance was recorded at 691 nm (λ_max_ of pyocyanin) [29] using a microplate reader (VERSA max, Bio-Strategy Pty Ltd, Australia).

H_2_O_2_ generation in the LB medium mediated by phenazine production by various *P. aeruginosa* strains at various growth times was measured using a colorimetric assay [17]. To a freshly prepared solution of 160 µl of sodium acetate (0.1 M) containing 0.1 µg of horseradish peroxidase (Thermo Scientific) and 10 µl of 1 mg/ml of o- dianisidine (Alfa Aesar) in methanol (Unichrom, Ajax Finechem Pty Ltd, Australia), 40 µl of the bacterial cell free supernatants were added in 96-well microtiter plates and incubated for 10 min at room temperature protected from light. The absorbance of H_2_O_2_ in the mixture solution was determined using a microplate reader (VERSA max, Bio-Strategy Pty Ltd, Australia) at 570 nm. For standards commercially available 30% H_2_O_2_ (Univar, USA) was diluted in LB medium to 0.01%. The absorbance of 0.01% H_2_O_2_ (absorbance = 0.11 at 570 nm) is measured as described above by mixing 40 µl 0.01% H_2_O_2_ with 160 µl of mixture solution.

### Quantification of Bacterial Growth and Bacterial Cell Lysis

The influence of pyocyanin production in the growth of various *P. aeruginosa* strains in LB medium were monitored over 120 h. The number of bacterial cells/ml at various growth times (0, 8, 16, 24, 72 and 120 h) were recorded directly using a Bio-Rad Smartspec 3000 (Bio-Rad Laboratories Pvt Ltd, USA) at 600 nm using plain LB medium without bacterial cells as blank. The influence of pyocyanin production in bacterial cell lysis was quantified as percentage of cells alive according to:

(Max. number of cells/ml – number of cells/ml after growth time) × 100%.

### H_2_O_2_ Treatment to the 24 h Grown PAO1 Wildtype and PA14 ΔphzA-G Strains of *P. aeruginosa*

To 24 h grown planktonic cultures of PAO1 wildtype and PA14 Δ*phz A-G* strains of *P. aeruginosa,* final concentrations of 0, 0.01, 0.1 and 1% H_2_O_2_ were added and incubated for various times (0, 4, 16 and 24 h) at 30°C at 150 rpm. Extracellular DNA release was analysed using a Qubit 2.0 Fluorometer as described above. In addition, the change in number of bacterial cells and percentage of cells alive due to H_2_O_2_ was monitored after each incubation time using Bio-Rad Smartspec 3000 at OD 600 nm as described above.

### Statistical Analysis

The absorbance of pyocyanin produced by various *P. aeruginosa* strains and its influence on eDNA release, H_2_O_2_ generation, number of cells and percentage of cells alive were analyzed using a two-tailed Student’s t-test. A similar analysis was carried out for eDNA release mediated by exogenous addition of pyocyanin and eDNA release, number of cells and percentage of cells alive mediated by exogenous addition of H_2_O_2._ Differences were considered significant if p<0.05.

## Results

### Pyocyanin Production and eDNA Release in *P. aeruginosa* Over Time

Pyocyanin production and eDNA release were quantified in pyocyanin producing and pyocyanin deficient strains of *P. aeruginosa*.

**Figure 2:**
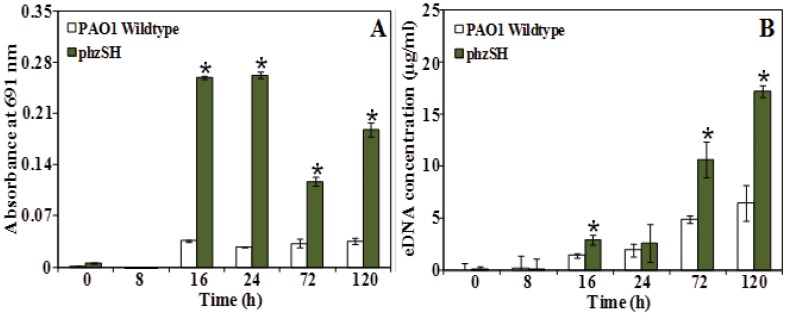
Production of pyocyanin and eDNA release in *P. aeruginosa* PAO1 strains. Pyocyanin absorbance (A) and eDNA concentration (B) in supernatants of *P. aeruginosa* PAO1 wildtype and *phzSH* mutant over time. Error bars represents standard deviations from the mean (n = 3). Asterisks indicate statistically significant (p<0.05) differences in absorbance and eDNA concentration in comparison to the PAO1 wildtype.

Figure 2 compares the pyocyanin and eDNA concentration in batch cultures of wildtype *P. aeruginosa* PAO1 and a *phzSH* mutant. The *phzSH* mutant which is deficient of *phzS* and *phzH* genes was unable to convert PCA to PCN or 1-OHPHZ [21], [23] and consequently overproduces pyocyanin (Fig. 2A). In accordance with the hypothesis that pyocyanin is involved in eDNA production the pyocyanin over producing *phzSH* mutant strain of *P. aeruginosa* PAO1 released significantly more eDNA that the wildtype strain after 16, 72 and 120 hours incubation (Fig. 2B).

**Figure 3:**
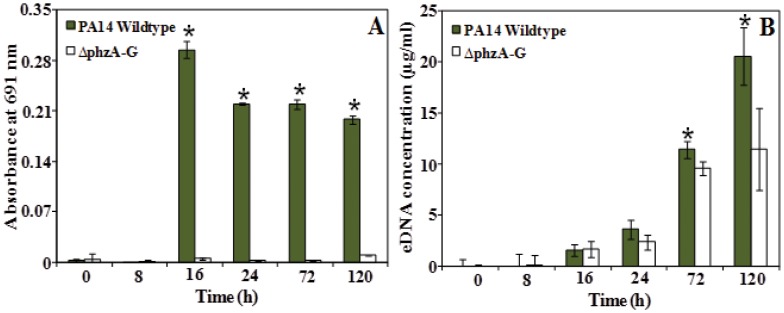
Production of pyocyanin and eDNA release in *P. aeruginosa* PA14 strains. (A) Pyocyanin absorbance (A) and eDNA concentration (B) in supernatants of *P. aeruginosa* PA14 wildtype and Δ*phzA-G* mutant over time. Error bars represent standard deviation from the mean (n = 3). Asterisks indicate statistically significant (p<0.05) differences in absorbance and eDNA concentrationin comparison to the mutant strain Δ*phzA-G*.

Figure 3 compares the pyocyanin and eDNA concentration in batch cultures of wildtype *P. aeruginosa* PA14 and a Δ*phzA-G* mutant. The Δ*phzA-G* mutant is incapable of producing PCA and consequently does not produce pyocyanin (Fig. 3A). In accordance with the hypothesis that pyocyanin is involved in eDNA production the pyocyanin deficient strain produced significantly less eDNA after 72 and 120 hours incubation (Fig. 3B).

It should be noted that pyocyanin and eDNA concentrations are not directly correlated. For example, the wildtype and *phzSH P. aeruginosa* PAO1 cultures displayed significant differences in pyocyanin concentration after 24 hours incubation yet there was no significant difference in eDNA concentration. The same is true of the wildtype and Δ*phzA-G* cultures of *P. aeruginosa* PA14 cultures after 16 and 24 h incubation. These observations suggest that if pyocyanin controls eDNA concentration then it does so indirectly via an intermediate agent.

### eDNA Release in *P. aeruginosa* in Response to Exogenous Pyocyanin Addition

**Figure 4:**
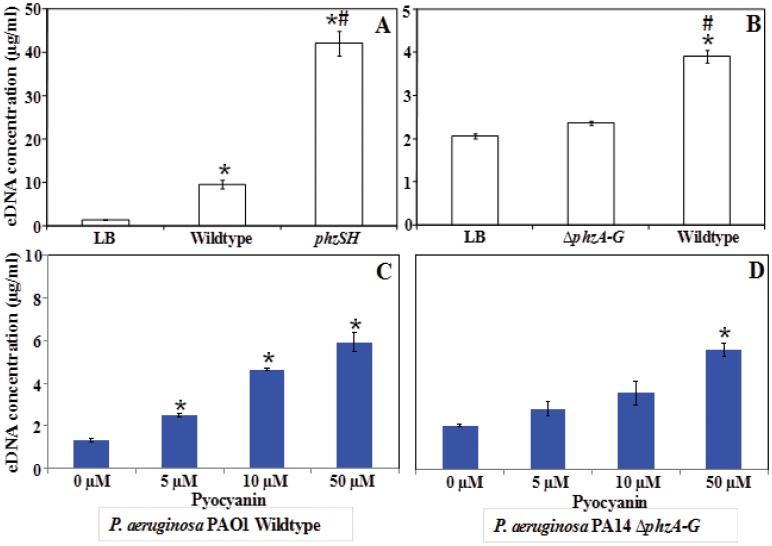
eDNA release in *P. aeruginosa* in response to exogenous supernatant or pyocyanin addition. (A) *P. aeruginosa* PAO1 wildtype grown for 24 h in the presence of supernatant from a 72 h old grown bacterial cell free supernatant of PAO1 wildtype and *phzSH* strains whereas and (B) *P. aeruginosa* PA14 Δ*phzA-G* grown in the presence of PA14 Δ*phzA-G* and wildtype strains. Both PAO1 wildtype and PA14 Δ*phzA-G* were also grown in the presence of LB broth. PAO1 wildtype (C) and PA14 Δ*phzA-G* (D) grown for 24 h as a function of concentration of pyocyanin. Error bars represent standard deviation from the mean (n = 3). Asterisks indicate statistically significant (p<0.05) differences in eDNA concentration in comparison to LB (A and B) and 0 µM pyocyanin (C and D). A hash indicates a statistically significant (p<0.05) difference in eDNA concentration in comparison to the wildtype (A) and Δ*phzA-G* mutant (B).

To test whether a component in the supernatant of pyocyanin producing cultures was responsible for the increase in eDNA release, *P. aeruginosa* PAO1 wildtype and the PA14 Δ*phzA-G* mutant were cultured in the presence of 72 h culture supernatants from *P. aeruginosa* PAO1 *phzSH* and PA14 wildtype respectively. Figure 3 illustrates a significant increase in eDNA concentration in response to the pyocyanin laden supernatants. Supernatants from strains with low or moderate pyocyanin production and fresh LB media did not stimulate eDNA release (Fig. 4). To investigate the impact of exogenously added pyocyanin on eDNA concentration directly, *P. aeruginosa* PAO1 wildtype and the PA14 Δ*phzA-G* mutant cultures were monitored over 24 hours in the presence of 0, 5, 10 or 50 µM pyocyanin. Figure 4 shows a clear dose response relationship between eDNA release and pyocyanin concentration in both cultures.

### Pyocyanin Mediated H_2_O_2_ Generation and Cells Lysis

**Figure 5:**
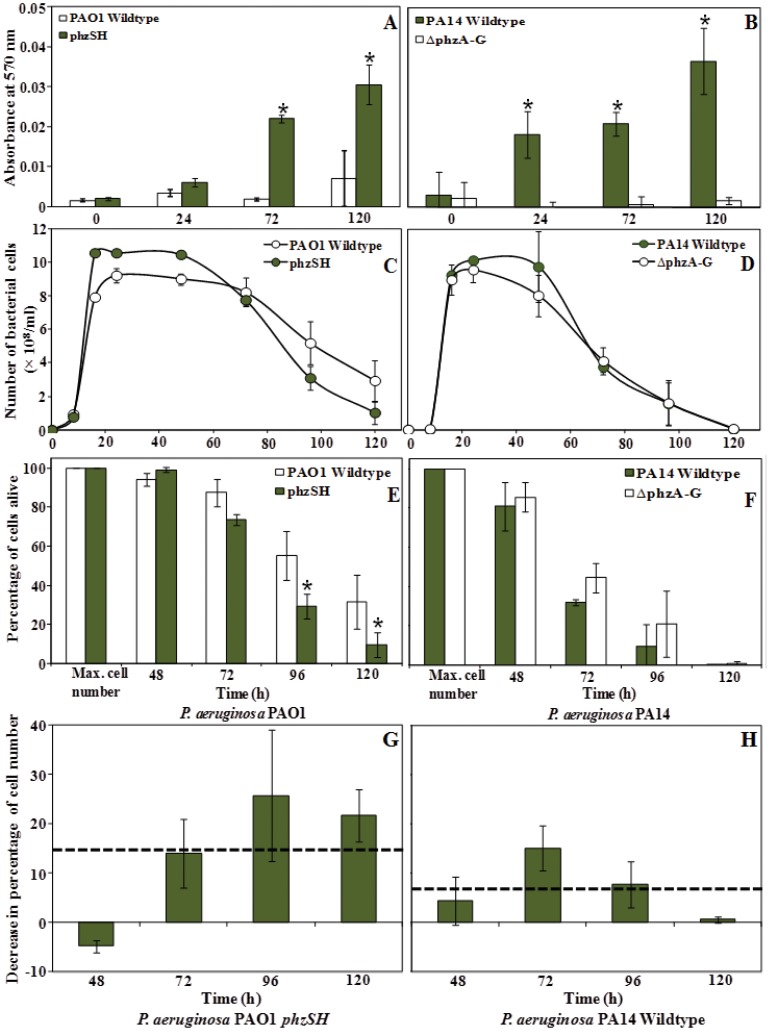
Pyocyanin mediated H_2_O_2_ generation and cells lysis. Increased H_2_O_2_ production as recorded in bacterial cell free supernatant in PAO1 mutant *phzSH* (A) and in PA14 wildtype (B) compared with strains producing less pyocyanin. The *P. aeruginosa* PAO1 (C, E and G) and PA14 (D, F and H) strains producing more pyocyanin (PAO1 *phzSH* mutant and PA14 wildtype) displayed accelerated decreases in optical density as cultures aged compared with pyocyanin deficient strains. Error bars represent standard deviation from the mean (n = 3). Asterisks indicate statistically significant (p<0.05) differences in H_2_O_2_ absorbance in comparison to the PAO1 wildtype and PA14 *phzA-G* mutant (A and B) and percentage of cells alive in comparison to max. cell number (E and F).Dashed line indicates the average decrease in percentage of cell number due to H_2_O_2_ generation in PAO1 *phzSH* and PA14 wildtype strains (G and H).

A colorimetric assay for H_2_O_2_ quantification was used to follow H_2_O_2_ generation in wildtype and mutant strains of PAO1 and PA14 producing modest and excessive quantities of pyocyanin. Figure 5 demonstrates that the strains producing the highest concentrations of pyocyanin generated the highest concentrations of H_2_O_2_.

To relate H_2_O_2_ production to cell lysis the optical density of the cultures was also monitored over time (Fig 5C and D). The optical density of all cultures decreased over time reflecting H_2_O_2_ independent mechanisms of cell lysis in *P. aeruginosa* PAO1 and PA14 (Fig. 5C to F). Despite this background drop in cell density, an additional decrease in optical density on average of about 14% for PAO1 *phzSH* and 7% for PA14 wildtype strains was clearly discernable in the prodigious pyocyanin producing cultures that were generating higher peroxide concentrations (Fig. 5G and H).

### Exogenous H_2_O_2_ Addition Results in a Decrease in Bacterial Cell Number and eDNA Release

**Figure 6:**
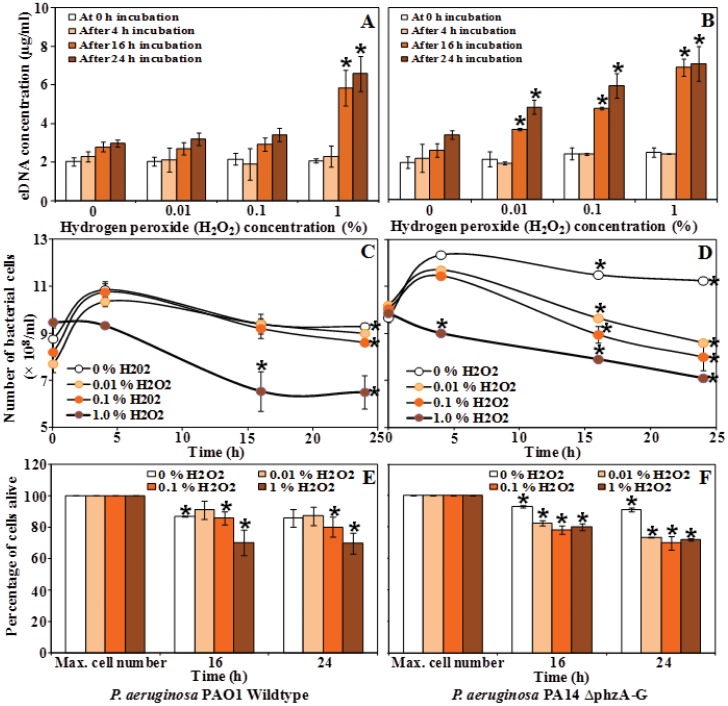
Exogenous H_2_O_2_ addition results in a decrease in bacterial cell number and increased eDNA release. (A) Dose dependent H_2_O_2_ treatment shows a significant increase in eDNA concentration in bacterial cell free supernatant for PAO1 wildtype especially after 16 and 24 h incubation with 1% H_2_O_2_. (B) The PA14 Δ*phzA-G* mutant showed significant increases in eDNA at all H_2_O_2_ concentration after 16 and 24 h incubation. (C-F) Decrease in bacterial cell number and decrease in cell number (%) due to cell lysis mediated by H_2_O_2_ treatment. Error bars represent standard deviation from the mean (n = 3). Asterisks indicate a statistically significant (p<0.05) difference in eDNA concentration in comparison to 0% concentration H_2_O_2_ treatment (A and B) and in number of bacterial cells in comparison to the max. cell number (×10^8^/ml) as a function of time (C and D) and percentage of cells alive in comparison to the max. cell number (E and F).

To examine the relationship between H_2_O_2_ concentration and eDNA release in *P. aeruginosa* PAO1 and PA14, the PAO1 wildtype and the PA14 Δ*phzA-G* mutant were incubated in the presence of 0, 0.01, 0.1 and 1% v/v H_2_O_2_ and monitored over 24 hours. A decrease in bacterial cell number was observed for the PAO1 wildtype at 1% H_2_O_2_ v/v as measured by optical density. Interestingly, the *P. aeruginosa* PA14 Δ*phzA-G* mutant was more sensitive showing significant decreases in optical density at 0.01, 0.1 and 1% v/v H_2_O_2_ (Fig. 6). Congruently, 1% v/v H_2_O_2_ caused a significant increase in eDNA concentration in the *P. aeruinosa* PAO1 cultures after 16 and 24 h incubation (Fig. 6) and the *P. aeruginosa* PA14 Δ*phzA-G* mutant showed significant increases in eDNA concentration with H_2_O_2_ concentrations 0.01% and above after 16 and 24 h incubation (Fig. 6).

## Discussion

*P. aeruginosa* produces a variety of phenazines which were formerly disregarded as bacterial secondary metabolites but have recently garnered much attention and been ascribed a variety of roles in microbial ecology. In the context of pathogenic interactions with epithelial cells of human airways, phenazines oxidise glutathione and NADH leading to loss of redox homeostasis in host cells resulting in cell injury or cell death [22]. In other environments such as soil, phenazines are toxic to competing organisms including bacteria and fungi presumably through production of reactive oxygen species [22].

Pyocyanin has been implicated as a physiological signal interfacing with quorum sensing regulatory networks influencing expression of genes involved in efflux pumps, redox homeostasis and iron acquisition [30]. It has also been linked to respiration through extracellular electron transfer enabling survival of cells in oxygen depleted environments such as thick biofilms, thereby influencing colony and biofilm morphology [31]. In this study evidence is presented supporting the hypothesis that the phenazine pyocyanin also impacts on extracellular DNA concentrations in planktonic *P. aeruginosa* populations through H_2_O_2_ generation and subsequent cell lysis.

Two distinct strains of *P. aeruginosa* were used in this study to examine the role of pyocyanin in determining eDNA concentrations. The first strain (PAO1) rations transformation of the central phenazine PCA into three distinct phenazines, of which one is pyocyanin, and consequently produces relatively low quantities of pyocyanin [23]. The second strain (PA14) preferentially diverts PCA into pyocyanin production and therefore produces pyocyanin prodigiously. A *phzSH* mutant of PAO1 which produces higher quantities of pyocyanin than its wildtype and a Δ*phzA-G* mutant of PA14 which produces relatively little pyocyanin were also used. By examining pyocyanin production in these four strains in relation to H_2_O_2_ generation, cell lysis and eDNA concentration a clear relationship emerged.

In both PAO1 and PA14 strains, pyocyanin production peaked between 16 and 24 h batch culture growth whilst concentrations decreased over 72–120 h incubation (Fig. 2A and 3A). The production of H_2_O_2_ was observed to coincide with the decrease in pyocyanin production (Fig. 5A and B). The rise and fall in pyocyanin concentration is known to be a consequence of redox cycling in pyocyanin, which generates reactive oxygen species such as H_2_O_2_ [22] that subsequently oxidatively degrades pyocyanin [29]. The rise in H_2_O_2_ concentrations coincided with an increase in cell lysis and eDNA release (Fig. 2B, 3B, and 5). H_2_O_2_ generation is known to impart oxidative stress resulting in cell lysis in bacteria [17] and cell lysis is a well known source of eDNA release [16], [17], [19]. However *P. aeruginosa* PAO1 encodes resistance to basal levels of oxidative stress caused by basal levels of pyocyanin mediated hydrogen peroxide production, through catalase activity that enhances the disproportionation of hydrogen peroxide into water and oxygen [32]. As pyocyanin production increased in the PAO1 *phzSH* mutant subsequent increases in oxidative stress as a consequences of hydrogen peroxide generation resulted in an additional 14% increase in cell lysis and significant increase in eDNA release.

Further experiments were carried out in which spent *P. aeruginosa* supernatants from pyocyanin producing strains stimulated eDNA release in fresh *P. aeruginosa* cultures (Fig. 4A and B). Exogenous addition of synthetic pyocyanin was also shown to mimic the effect of the supernatant addition, resulting in increases in eDNA concentration (Fig. 4C and D). Finally, exogenous H_2_O_2_ addition was confirmed to result in increased cell lysis and eDNA concentration (Fig. 6).

Despite being an obvious disadvantage to individual cells, cell lysis has emerged as an essential process in the biofilm lifecycle [33]. Specifically, PQS mediates bacterial cell death and release of eDNA in relatively young biofilms thereby enhancing biofilm stability under hostile environmental conditions [34]. In the case of older biofilms flagella and pilli mediated cell lysis promotes cell dispersal and ultimately establishment of new colonies [20]. In conflict with these ideas, *P. aeruginosa* PA14 phage mediated cell lysis inhibits biofilm formation and swarming motility [35].

The findings in this study demonstrate that pyocyanin is involved in eDNA release in growing *P. aeruginosa* planktonic cultures raising interesting questions about its role in biofilm biology. Pyocyanin mediated eDNA production, which likely happens as a consequence of cell lysis via H_2_O_2_ generation, could possibly assist *P. aeruginosa* biofilm formation in several ways, since eDNA has been proven to be essential at all stages of biofilm formation. Previous studies suggest that the production of phenazines enhance bacterial adhesion, microcolony formation and increased biomass in biofilms of *P. aeruginosa* [25], [27], [36]. From the previous findings in concurrence with the current results, we propose that the phenazine pyocyanin may promote biofilm formation in *P. aeruginosa* via eDNA release through H_2_O_2_ mediated cell lysis. In addition, we also hypothesize that pyocyanin could possibly promote eDNA release in other bacterial species that persist in mixed biofilm along with *P. aeruginosa* and henceforth may have significant ecological impacts on mixed biofilms.
